# Incidence and severity of transient hypothyroxinaemia of prematurity associated with survival without composite morbidities in extremely low birth weight infants

**DOI:** 10.1038/s41598-019-46108-9

**Published:** 2019-07-03

**Authors:** Shin Ae Yoon, Yun Sil Chang, So Yoon Ahn, Se In Sung, Won Soon Park

**Affiliations:** 1 Department of Paediatrics, Chungbuk National University Hospital, Cheongju, Korea; 2 Department of Paediatrics, Samsung Medical Center, Sungkyunkwan University School of Medicine, Seoul, Korea

**Keywords:** Thyroid diseases, Neonatology, Preterm birth, Paediatric research

## Abstract

This study investigated the incidence of transient hypothyroxinaemia of prematurity (THOP) associated with survival without composite morbidities and the predictability of THOP severity in extremely low birth weight infants (ELBWIs). We retrospectively reviewed the medical records of 546 ELBWIs who underwent initial thyroid function tests within 14 postnatal days, with 156 ELBWIs from 2000 to 2005 (period I) and 390 from 2006 to 2013 (period II). The infants were stratified into 23–24, 25–26 and 27–28 weeks’ gestation subgroups within each period; the initial thyroxine (T4) level, mortality, clinical characteristics and composite morbidities, including bronchopulmonary dysplasia, intraventricular haemorrhage, necrotizing enterocolitis, and retinopathy of prematurity were analysed. The predictive value of the initial T4 level, Apgar score at 5 min, and clinical risk index for babies II (CRIB II) score for estimating mortality and survival with or without composite morbidities was assessed. Comparing period II and period I, the incidence of THOP was significantly decreased along with significantly increased survival without composite morbidities in ELBWIs at 25–28 weeks’ gestation. The severity of THOP showed significant associations with mortality and composite morbidities. The initial T4 level was most effective for predicting outcome compared with Apgar and CRIB II scores.

## Introduction

Transient hypothyroxinaemia of prematurity (THOP), characterised by low thyroxine (T4) levels with normal or low thyroid stimulating hormone levels, occurs in more than half of the extremely low birth weight infants (ELBWIs) born at <30 weeks^1,2^. The low T4 levels reach a nadir at 7–10 days postnatal and could remain low for the first 3–6 weeks of the postnatal period, depending on the extent of prematurity^3,4^. Although the aetiologies of THOP are multifactorial, including the loss of maternal placental transfer of T4 after birth, immaturity of the hypothalamus-pituitary-thyroid axis, limited thyroid capacity to increase synthesis, and metabolism and drugs affecting thyroid function^5^, recent studies have demonstrated that THOP is associated with increases in perinatal mortality and morbidity^6^; prolonged mechanical ventilation^7^; and non-thyroidal illnesses such as respiratory distress syndrome (RDS)^3,8^, intraventricular haemorrhage (IVH)^9,10^, and cerebral white matter damage^11^. Furthermore, in concordance with the adult data on non-thyroidal illness, the illness severity in premature infants seemed to be an important determinant of low serum T4 levels^12,13^. Collectively, these findings suggest that THOP might just be the epiphenomenon reflecting the severity of the non-thyroidal illness in ELBWIs.

Recently, we reported improved survival rate and increased survival rates without composite morbidities such as bronchopulmonary dysplasia (BPD), severe IVH, necrotizing enterocolitis (NEC), and retinopathy of prematurity (ROP) with better perinatal and neonatal intensive care for the ELBWIs^14–16^. However, the temporal alterations in the incidence of THOP associated with the recently improved survival rates without composite morbidities of ELBWIs have not been studied yet. Furthermore, although prediction of mortality and morbidities in ELBWIs prior to discharge is of the utmost clinical importance, the predictability of THOP severity status for mortality, morbidities and survival without composite morbidities has not been tested yet. Therefore, in the present study, we investigated whether survival without composite morbidities was associated with the altered incidence of THOP, and if applicable, the potential of THOP severity to predict outcome for ELBWIs. Specifically, we compared the predictive power of the initial T4 levels for estimating mortality, composite morbidities, and survival without major morbidities in ELBWIs with that of the Apgar score at 5 min and clinical risk index for babies II (CRIB II) score. The Apgar and CRIB II scores were found to be good predictors of mortality in our previous study^17^.

## Results

### Clinical characteristics

Table 1 shows the demographic and clinical findings of the enrolled infants in each subgroup and study period. The total GA and birth weight were significantly lower and antenatal steroid use was significantly higher, especially in the infants at 23–24 weeks’ gestation, during period II than in period I. The total Apgar scores at 1 min and 5 min of infants with a gestational age of 23–28 weeks were significantly higher during period II than during period I. Other variables including sex, SGA, and caesarean section did not differ significantly between infants enrolled in the 2 study periods.

**Table 1:** Clinical characteristics of enrolled infants in each subgroup and study period.

Variable	23–24 weeks (n = 173)		25–26 weeks (n = 246)		27–28 weeks (n = 127)		Total (n = 546)	
	P I (n = 36)	P II (n = 137)	P I (n = 73)	P II (n = 173)	P I (n = 47)	P II (n = 80)	P I (n = 156)	P II (n = 390)
Gestational age (weeks)	23.8 ± 0.4	23.6 ± 0.5^*^	25.5 ± 0.5	25.4 ± 0.5	27.3 ± 0.5	27.4 ± 0.5	25.7 ± 1.3	25.2 ± 1.5^*^
Birth weight (g)	683 ± 114 (414–900)	633 ± 99^*^ (370–860)	817 ± 123 (439–990)	805 ± 133 (380–990)	841 ± 107 (563–991)	819 ± 150 (380–990)	793 ± 131 (414–991)	747 ± 151^*^ (370–990)
Male	18 (50%)	67 (49%)	39 (53%)	80 (46%)	16 (34%)	44 (55%)^*^	73 (47%)	191 (49%)
One-minute Apgar score	3 ± 1	4 ± 1^*^	3 ± 2	5 ± 2^*^	4 ± 2	5 ± 1^*^	3 ± 2	5 ± 2^*^
Five-minute Apgar score	6 ± 2	7 ± 1^*^	6 ± 2	7 ± 1^*^	7 ± 1	8 ± 1^*^	6 ± 2	7 ± 1^*^
C/sec	26 (72%)	110 (80%)	51 (70%)	136 (79%)	39 (83%)	68 (85%)	116 (74%)	314 (81%)
SGA	4 (11%)	8 (6%)	4 (5%)	18 (10%)	14 (30%)	31 (39%)	22 (14%)	57 (15%)
Antenatal steroids	24 (67%)	117 (85%)^*^	53 (73%)	141 (82%)	35 (74%)	61 (76%)	112 (72%)	319 (82%)^*^
Chorioamnionitis	19 (53%)	79 (58%)	31 (43%)	82 (47%)	17 (36%)	28 (35%)	67 (43%)	189 (48%)
PIH	2 (6%)	4 (3%)	11 (15%)	17 (10%)	17 (36%)	24 (30%)	30 (19%)	45 (12%)^*^
GDM	1 (3%)	5 (4%)	2 (3%)	8 (5%)	1 (2%)	1 (1%)	4 (3%)	14 (4%)

^*^P < 0.05.

Values are presented as means ± SD (range) or n (%).

C/sec, caesarean section; SGA, small for gestational age; PIH, pregnancy-induced hypertension; GDM, gestational diabetes mellitus; SD, standard deviation.

### Changes in mortality, morbidities, and survival without major morbidity rates

Table 2 demonstrates the clinical outcomes for enrolled infants in each subgroup and study period. While the total mortality rate did not differ significantly during the study periods, the total BPD rate was significantly lower, the IVH rate was significantly higher and the survival without composite morbidities rate in the infants at 25–26 and 27–28 weeks’ gestation was significantly higher during period II than during period I.

**Table 2:** Clinical outcomes of enrolled infants in each subgroup and study period.

	23–24 weeks (n = 173)		25–26 weeks (n = 246)		27–28 weeks (n = 127)		Total (n = 546)	
	P I (n = 36)	P II (n = 137)	P I (n = 73)	P II (n = 173)	P I (n = 47)	P II (n = 80)	P I (n = 156)	P II (n = 390)
Mortality	10 (28%)	23 (17%)	4 (5%)	13 (8%)	2 (4%)	6 (8%)	16 (10%)	42 (11%)
Composite morbidities	28/35 (80%)	115/133 (86%)	57/73 (78%)	104/170 (61%)^*^	28 (60%)	33 (41%)^*^	113/155 (73%)	252/383 (66%)
BPD (≥moderate)	24 (67%)	74 (54%)	46 (63%)	67 (39%)^*^	20 (43%)	30 (38%)	90 (58%)	171 (44%)^*^
IVH (Gr ≥ 3)	7 (19%)	45 (33%)	6 (8%)	21 (12%)	0 (0%)	4 (5%)	13 (8%)	70 (18%)^*^
NEC (≥3b)	1/35 (3%)	8/135 (6%)	3/70 (4%)	7/173 (4%)	1 (2%)	0 (0%)	5/152 (3%)	15/388 (4%)
ROP requiring laser therapy	15/35 (43%)	70/129 (54%)	27/73 (37%)	46/166 (27%)	10/47 (21%)	6/79 (8%)^*^	52/155 (34%)	122/374 (33%)
Without composite morbidities	4 (11%)	18 (13%)	15 (21%)	66 (38%)^*^	18 (38%)	46 (58%)^*^	37 (24%)	130 (33%)^*^

^*^P < 0.05.

BPD, bronchopulmonary dysplasia; IVH, intraventricular haemorrhage; NEC, necrotizing enterocolitis; ROP, retinopathy of prematurity.

Composite morbidities = BPD (≥moderate) + IVH (Gr ≥ 3) + NEC (≥3b) + ROP requiring laser therapy.

### Changes in the initial thyroxine levels

Table 3 shows the alterations in the total and fT4 levels between enrolled infants in each subgroup and study period. While the incidence of THOP in each subgroup did not differ significantly between the 2 study periods, the incidence of normal initial thyroxine levels (T4 ≥ 4.5 ng/dl and/or fT4 ≥ 0.9 ng/dl) in the infants at 25–26 weeks’ and 27–28 weeks’ gestation during period II was significantly higher than during period I.

**Table 3:** Initial total and/or free thyroxine levels between enrolled infants in each subgroup and study period.

	23–24 weeks (n = 173)		25–26 weeks (n = 246)		27–28 weeks (n = 127)		Total (n = 546)	
	P I (n = 36)	P II (n = 137)	P I (n = 73)	P II (n = 173)	P I (n = 47)	P II (n = 80)	P I (n = 156)	P II (n = 390)
T4 < 2.5 ng/dl and/or f T4 < 0.5 ng/dl	22 (61%)	74 (54%)	20 (27%)	38 (22%)	8 (17%)	8 (10%)	50 (32%)	120 (31%)
2.5 ≤ T4 < 4.5 ng/dl and/or 0.5 ≤ fT4 < 0.9 ng/dl	9 (25%)	49 (36%)	37 (51%)	71 (41%)	20 (43%)	23 (29%)	66 (42%)	143 (37%)
T4 ≥ 4.5 ng/dl and/or fT4 ≥ 0.9 ng/dl	5 (14%)	14 (10%)	16 (22%)	64 (37%)^*^	19 (40%)	49 (61%)^*^	40 (26%)	127 (33%)

*P < 0.05.

T4, thyroxine; fT4, free thyroxine.

### Clinical outcomes according to the initial thyroxine level

The clinical outcomes of the infants were dependent on the initial T4 levels, with the highest mortality and composite morbidities rates and lowest survival without morbidities rates observed when initial T4 levels of <2.5 ng/dl and/or f T4 < 0.5 ng/dl, and the lowest mortality and composite morbidities rates and highest survival without morbidities rates observed when the initial T4 levels were ≥4.5 ng/dl and/or fT4 ≥ 0.9 ng/dl (Table 4). In binary logistic regression analyses with adjustment of gestational age, birth weight, sex and antenatal steroid use, the odds ratios of composite morbidities including BPD (≥moderate) and IVH (Gr ≥ 3) were significantly increased, and the odds ratio of without composite morbidities was significantly reduced in initial T4 levels of <4.5 ng/dl and/or f T4 < 0.9 ng/dl, but the odds ratios of mortality and ROP requiring laser therapy was significantly increased only in initial T4 levels of <2.5 ng/dl and/or f T4 < 0.5 ng/dl (Table 5).

**Table 4:** Clinical outcomes according to the initial total and/or free thyroxine levels

	T4 < 2.5 ng/dl and/or f T4 < 0.5 ng/dl^a^ (n = 170)	2.5 ≤ T4 < 4.5 ng/dl and/or 0.5 ≤ fT4 < 0.9 ng/dl^b^ (n = 209)	T4 ≥ 4.5 ng/dl and/or fT4 ≥ 0.9 ng/dl^c^ (n = 167)	*P*-value
Mortality	37 (22%)	17 (8%)	4 (2%)	<0.001^a<b<c^
Composite morbidities	137/163 (84%)	152/208 (73%)	76/167 (46%)	<0.001^a, b<c^
BPD (≥moderate)	97 (57%)	109 (52%)	55 (33%)	<0.001^a,b<c^
IVH (Gr ≥ 3)	43 (25%)	33 (16%)	7 (4%)	<0.001^a<b<c^
NEC (≥3b)	9/167 (5%)	9/206 (4%)	2/167 (1%)	0.0427
ROP requiring laser therapy	78/156 (45%)	67/207 (32%)	29/166 (17%)	<0.001^a<b<c^
Without composite morbidities	24 (14%)	54 (26%)	89 (53%)	<0.001^a<b<c^

T4, thyroxine; fT4, free thyroxine; BPD, bronchopulmonary dysplasia; IVH, intraventricular haemorrhage; NEC, necrotizing enterocolitis; ROP, retinopathy of prematurity.

Composite morbidities = BPD (≥moderate) + IVH (Gr ≥ 3) + NEC (≥3b) + ROP requiring laser therapy.

**Table 5:** Results of binary logistic regressions on clinical outcomes according to the initial total and/or free thyroxine levels.

	T4 < 2.5 ng/dl and/or f T4 < 0.5 ng/dl (n = 170)		2.5 ≤ T4 < 4.5 ng/dl and/or 0.5 ≤ fT4 < 0.9 ng/dl (n = 209)		T4 ≥ 4.5 ng/dl and/or fT4 ≥ 0.9 ng/dl (n = 167)
	OR (95% CI)	*P*-value	OR (95% CI)	*P*-value	Reference
Mortality	5.018 (1.560–16.140)	0.007	2.785 (0.873–8.892)	0.084	1
Composite morbidities	2.976 (1.663–5.327)	<0.001	2.563 (1.619–4.058)	<0.001	1
BPD (≥moderate)	1.981 (1.187–3.307)	0.009	2.028 (1.305–3.153)	0.002	1
IVH (Gr ≥ 3)	3.249 (1.309–8.065)	0.011	2.850 (1.193–6.811)	0.018	1
NEC (≥3b)	2.109 (0.965–4.611)	0.061	1.245 (0.592–2.615)	0.564	1
ROP requiring laser therapy	2.244 (1.258–4.011)	0.006	1.563 (0.926–2.640)	0.095	1
Without composite morbidities	0.327 (0.180–0.593)	<0.001	0.386 (0.242–0.615)	<0.001	1

Data are adjusted for gestational age, birth weight, sex and antenatal steroid use.

T4, thyroxine; fT4, free thyroxine; BPD, bronchopulmonary dysplasia; IVH, intraventricular haemorrhage; NEC, necrotizing enterocolitis; ROP, retinopathy of prematurity.

Composite morbidities = BPD (≥moderate) + IVH (Gr ≥ 3) + NEC (≥3b) + ROP requiring laser therapy.

### ROC curves of variables for predicting mortality, composite morbidities, and intact survival

**Figure 1:**
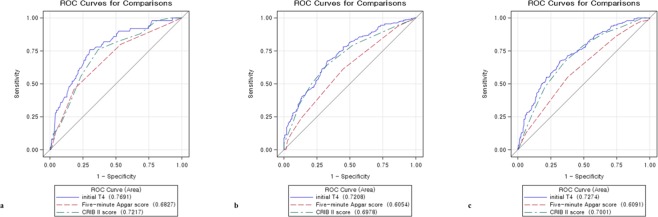
ROC curves of the initial thyroxine level, Apgar score at 5 min, and clinical risk index for babies II score for predicting mortality (**a**), composite morbidity (**b**), and intact survival (**c**).

Figure 1 and Table 6 demonstrate the ROC curves of the initial T4 level, Apgar, and CRIB II scores for predicting mortality, composite morbidities, and survival without morbidities, respectively. The initial T4 level was a better predictor for mortality (cut-off value 2.55, AUC 0.769, sensitivity 76%, specificity 70%), composite morbidities (cut-off value 3.62, AUC 0.721, sensitivity 43%, specificity 83%), and survival without morbidities (cut-off value 3.62, AUC 0.727, sensitivity 16%, specificity 55%) than the CRIB II and Apgar scores.

**Table 6:** Screening analysis and values of the area under the ROC curve of initial thyroxine level, Apgar score at 5 min and clinical risk index for babies II score for predicting mortality, composite morbidity, and intact survival.

		AUC	SE	95% CI	Cut-off point	Sensitivity	Specificity	PPV	NPV
Mortality	Initial T4	0.769	0.035	0.701–0.837	≤2.55	76%	70%	24%	96%
	Five-minute Apgar score	0.683	0.039	0.606–0.760	≤6.5	50%	75%	19%	93%
	CRIB II score	0.722	0.036	0.651–0.792	≥13.5	29%	35%	5%	80%
Composite morbidities	Initial T4	0.721	0.025	0.671–0.770	≤3.62	43%	83%	84%	43%
	Five-minute Apgar score	0.605	0.027	0.553–0.660	≤7.5	31%	82%	78%	36%
	CRIB II score	0.700	0.026	0.647–0.750	≥12.5	54%	21%	59%	18%
Survival without morbidities	Initial T4	0.727	0.025	0.679–0.776	≥3.62	16%	55%	15%	58%
	Five-minute Apgar score	0.609	0.027	0.557–0.662	≥7.5	18%	68%	21%	65%
	CRIB II score	0.700	0.026	0.650–0.750	≤12.5	79%	47%	40%	83%

ROC, receiver operating characteristic; AUC, area under the curves; SE, standard error; CI, confidence interval; PPV, positive predictive value; NPV, negative predictive value; T4, thyroxine; CRIB, clinical risk index for babies.

Composite morbidities = BPD (≥moderate) + IVH (Gr ≥ 3) + NEC (≥3b) + ROP requiring laser therapy.

## Discussion

THOP has been known to be associated with increases in perinatal mortality and morbidities^10,12,18^. Recently, we reported significantly improved mortality and morbidities in extremely preterm (EPT) infants with better perinatal and neonatal intensive care^14–16^. In this study, while the total GA and birth weight were significantly lower, the Apgar scores at 1 and 5 min were significantly higher, and the incidence of normal TFTs in ELBWIs at 25–26 and 27–28 weeks’ gestation significantly increased from 22% and 40% during period I to 37% and 61% during period II respectively. This increase was in strict concordance with the significantly increased survival without composite morbidities from 21% and 38% during period I to 38% and 58% during period II, respectively. To the best of our knowledge, this is the first study that studied temporal changes in the incidence of THOP. The change may have been linked to the continuing improvement in perinatal and neonatal intensive care resulting in a better outcome for EPT infants. Collectively, these findings suggest that THOP might be an epiphenomenon of non-thyroidal illness in ELBWIs, and its incidence could thus be reduced by attenuating non-thyroidal illness with better clinical management in ELBWIs.

In the present study, the mortality, the prevalence of morbidities, composite morbidities and survival without morbidities was dependent on the initial T4 level, with the highest mortality and lowest survival without morbidities observed with the lowest T4 levels of less than 2.5 ng/dl. In concordance with our data, several studies have noted that the severity of the non-thyroidal illness is an important determinant of low serum T4 levels in premature infants^12,13,19–22^. Overall, these findings suggest that the initial T4 levels reflect the severity of the non-thyroidal illness and thus could predict the outcome in ELBWIs.

Thyroid hormone has been known to play a critical role in perinatal neural development including brain myelination and production and maturation of oligodendrocytes^23^. Therefore, despite its transient nature and the epiphenomenon of severe illness, hypothyroxinaemia in ELBWIs could be a risk factor by itself for both acute mortality and morbidities^10^ and later neurodevelopmental deficits in ELBWIs^24–26^. However, a recent study reported that GA, but not THOP, played a key role in neuromotor dysfunction in preterm babies^27^. Moreover, thyroid hormone treatment did not attenuate the severity of RDS^28,29^, reduce mortality up to the point of discharge^29^, or improve long-term neurodevelopmental outcome^28,29^. Overall, these findings suggest that other factors associated with acute non-thyroidal illness in premature infants, rather than hypothyroxinaemia itself, might play a pivotal role in negatively impacting brain development and subsequent neurodevelopmental outcome. Further studies strictly limiting the study cohort to ELBWIs with THOP would be necessary to determine the potential beneficial effects of T4 supplementation^10^.

Predicting the outcome of ELBWIs at the highest risk of mortality and morbidities would be very helpful for the difficult decision making regarding treatment^30^. In our previous study, the Apgar score at 5 min and the lowest serum albumin level were the best predictors of mortality during ≤7^th^ and >7^th^ postnatal days, respectively in ELBWIs^17^. In this study using the same data set, the initial T4 level showed the best predictability of mortality, composite morbidities, and survival without major morbidities compared with Apgar score at 5 min and the CRIB II score which was shown to be a good predictor for ELBWI mortality in our previous study^17^ As the initial T4 level reflects the severity of non-thyroidal illness, and is associated with the ensuing mortality and morbidities in ELBWIs in the present and other studies^12,13,19–22^, these findings suggest that an increased initial T4 level might best reflect recent improvements in clinical management and the resultant better outcome for ELBWIs, and thus could be most effective for predicting their prognosis compared with the currently used CRIB II and Apgar scores. Accordingly, further studies will be necessary to develop new and effective models for better prediction of prognosis in ELBWIs.

The present study had several limitations including its retrospective and uncontrolled observational study design. We determined mortality and composite morbidities only at hospital discharge. Therefore, it was not apparent whether there was any causal relationship between the initial T4 levels and mortality and/or morbidities in these infants. Another limitation was that as our data were obtained from a single institution, the results obtained in this study might not be generalizable to other institutions. Nonetheless, we used a relatively large sample size of 546 ELBWIs born and admitted to a single centre with similar baseline clinical characteristics. Furthermore, although the consistency of clinical management was not measured, our data showing more antenatal steroid use and significantly higher Apgar scores at 1 and 5 min despite significantly lower GA and birth weight suggest that better perinatal and neonatal intensive care was provided during period II than during period I. Therefore, these findings imply that this single-centre study appropriately determined that the temporal alterations in the incidence and severity of THOP were associated with better clinical treatment and the ensuing improved outcomes in ELBWIs. These factors might also compensate for the limitations of the study.

In summary, the incidence of THOP was significantly reduced in ELBWIs at 25–28 weeks’ gestation during period II compared to period I, in parallel with their significantly higher Apgar scores, decreased morbidities and increased survival without composite morbidities. In ELBWIs, the initial T4 level was the best predictor of mortality, composite morbidities, and survival without composite morbidities in comparison to CRIB II and Apgar scores.

## Methods

### Ethics statement

The data collection procedure was approved by the Institutional Review Board of Samsung Medical Center (2018-09-024), and the Institutional Review Board waived the requirement for informed consent in this retrospective chart review.

### Primary outcome

Medical records of 546 ELBWIs (birth weights < 1,000 g) with gestational ages (GA) between 23 and 28 weeks who were born at and admitted to the Samsung Medical Center neonatal intensive care unit and had undergone initial thyroid function tests (TFTs) within the first 2 postnatal weeks, between January 2000 and July 2013 were retrospectively reviewed. Among them, 156 were born between January 1, 2000 and December 31, 2005 (period I), while 390 were born between January 1, 2006 and July 31, 2013 (period II). We arbitrarily divided the study period according to the survival rate of these ELBWIs as estimated in our previous studies^14–17^, and stratified them into the 23–24, 25–26 and 27–28 weeks’ gestation subgroups. We compared maternal and neonatal variables, survival and morbidity rates and incidence of thyroid dysfunction between the infants belonging to the 2 time periods.

### Data collection

Clinical characteristics, including GA, birth weight, Apgar scores at 1 and 5 min, sex, delivery mode, small for GA (SGA) (birth weight below the 10^th^ percentile), pregnancy-induced hypertension, gestational diabetes mellitus and antenatal steroid use, were analysed. GA was determined based on the maternal last menstrual period and the modified Ballard test. We analysed outcome measures, including death before discharge, BPD (≥moderate)^31^, IVH (≥grade 3)^32^, NEC (≥Bell’s stage 3b)^33^ and ROP requiring laser treatment.

For TFTs, we defined THOP as a temporarily low initial T4 level of <4.5 ng/dl and/or free T4 (fT4) level of <0.9 ng/dl with a TSH level of <20.0 µIU/ml and considered severe THOP to be having an initial T4 level of <2.5 ng/dl and/or fT4 level of <0.5 ng/dl. TFTs were followed up every 2–6 weeks until hospital discharge.

We calculated the CRIB II score for each infant using the following variables: sex, GA, birth weight, and base excess.

### Statistical analysis

Continuous variables were presented as means ± standard deviation and compared using the Student’s *t*-test or Mann-Whitney U test. Categorical variables were presented as percentages and frequencies and compared using the chi-square or Fisher’s exact test. We carried out binary logistic regression analyses with adjustment of gestational age, birth weight, sex and antenatal steroid use to obtain odds ratios of clinical outcomes according to the initial thyroxine level. The analyses of specificity and sensitivity of the Apgar score at 5 min, CRIB II score and initial T4 level were performed through receiver operating characteristic (ROC) curves, and the area under the curve (AUC) was conducted in order to define the differences with statistical significance in predicting mortality rate with selection of the most suitable cut-off point. SPSS version 19.09 (SPSS Inc., Chicago, IL, USA) was used for all statistical analyses, and a *P* < 0.05 was considered statistically significant.

## Data Availability

The data that support the findings of this study are available from the corresponding author (wonspark@skku.edu) upon reasonable request.
